# Pulmonary Sequelae in Patients After Recovery From Coronavirus Disease 2019: A Follow-Up Study With Chest CT

**DOI:** 10.3389/fmed.2021.686878

**Published:** 2022-01-13

**Authors:** Xuejiao Liao, Dapeng Li, Zhi Liu, Zhenghua Ma, Lina Zhang, Jingke Dong, Yirong Shi, Xiaowen Gu, Guangping Zheng, Ling Huang, Lijun Yuan, Jing Cao, Dan Shu, Xiangyi Yang, Qing He, Guobao Li, Zheng Zhang, Lei Liu

**Affiliations:** ^1^Department of Chronic Follow-Up, Shenzhen Third People's Hospital, Shenzhen, China; ^2^National Clinical Research Center for Infectious Disease, Institute of Hepatology, Shenzhen Third People's Hospital, Shenzhen, China; ^3^School of Medicine, The Second Affiliated Hospital, Southern University of Science and Technology, Shenzhen, China; ^4^Department of the Third Pulmonary Disease, Shenzhen Third People's Hospital, Shenzhen, China; ^5^Department of Radiology, Shenzhen Third People's Hospital, Shenzhen, China

**Keywords:** coronavirus disease 2019 (COVID-19), severe acute respiratory syndrome coronavirus 2 (SARS-CoV-2), pulmonary sequelae, follow-up, computed tomography

## Abstract

**Objective:** The pulmonary sequelae of coronavirus disease 2019 (COVID-19) have not been comprehensively evaluated. We performed a follow-up study analyzing chest computed tomography (CT) findings of COVID-19 patients at 3 and 6 months after hospital discharge.

**Methods:** Between February 2020 and May 2020, a total of 273 patients with COVID-19 at the Shenzhen Third People's Hospital were recruited and followed for 6 months after discharge. Chest CT scanning was performed with the patient in the supine position at end-inspiration. A total of 957 chest CT scans was obtained at different timepoints. A semi-quantitative score was used to assess the degree of lung involvement.

**Results:** Most chest CT scans showed bilateral lung involvement with peripheral location at 3 and 6 months follow-up. The most common CT findings were ground-glass opacity and parenchymal band, which were found in 136 (55.3%) and 94 (38.2%) of the 246 patients at 3 months follow-up, and 82 (48.2%) and 76 (44.7%) of 170 patients at 6 months follow-up, respectively. The number of lobes involved and the total CT severity score declined over time. The total CT score gradually increased with the increasement of disease severity at both 3 months follow-up (trend test *P* < 0.001) and 6 months follow-up (trend test *P* < 0.001). Patients with different disease severity represented diverse CT patterns over time.

**Conclusions:** The most common CT findings were ground-glass opacity and parenchymal bands at the 3 and 6 months follow-up. Patients with different disease severity represent diverse CT manifestations, indicating the necessary for long-term follow-up monitoring of patients with severe and critical conditions.

## Introduction

The coronavirus disease 2019 (COVID-19) caused by the severe acute respiratory syndrome coronavirus 2 (SARS-CoV-2) virus has become global pandemic (1). As of March 23, 2021, 122,536,880 confirmed cases of COVID-19, including 2,703,780 deaths, have been reported worldwide, with numbers continuing to climb every day (2, 3). Based on previous experience of other viral pulmonary infections, patients who recover from such diseases are often found to suffer long-term pulmonary consequences of their infection (4). Residual radiological or functional pulmonary damage have already been observed in some COVID-19 patients at the time of hospital discharge or at follow-up (5–9). However, little is known about the Computed tomography (CT) findings and lung function of COVID-19 during the longer follow-up period.

CT can provide valuable information about the diagnosis, progression, prognosis, and recovery of COVID-19 patients (10). In China, CT was widely used as a diagnostic tool for COVID-19 in the early stage of the outbreak. Numbers of studies described many chest CT characteristics of COVID-19 patients during hospitalization, suggesting that the most common findings were ground-glass opacity (GGO) lesions (11, 12). Subsequently, several studies have monitored the progression of the disease using CT (13, 14). Some studies identified the different stages of the disease using pulmonary changes found on chest CT scans from initial diagnosis until hospital discharge (15–17). Previous study reported persistent radiographic changes in a subgroup of patients with severe illness at 3, 6, 9, and 12 months following hospital discharge (18). However, CT findings and lung function among patients with different disease severity over a longer follow-up period still need to be investigated. Therefore, we assessed the chest CT characteristics of patients with COVID-19 at 3 and 6 months after discharge, including changes from admission to follow-up and by disease severity.

## Methods

### Study Population

Between January 11, 2020 and April 27, 2020, a total of 462 patients with COVID-19 were admitted to Shenzhen Third People's Hospital, which is the only hospital authorized by the Shenzhen City Government for the treatment of patients with COVID-19. All patients were diagnosed by a positive result on real-time reverse transcription polymerase chain reaction (RT-PCR). Clinical data of all patients at admission, including age, gender, and presence of a chronic disease, were collected retrospectively as baseline characteristics. According to the national guidelines for community-acquired pneumonia and the diagnosis and treatment plan for COVID-19 in China, disease severity was categorized into four groups based on symptoms, laboratory results, and imaging findings including mild, moderate, severe, and critical condition (19). In the hospitalization, 3 patients were died. A total of 273 patients who underwent at least one CT scan at 3 or 6 months follow-up were included in the final analysis. Written informed consent was obtained from all study participants and the study protocol was approved by the Ethics Committee of Shenzhen Third People's Hospital (2020-235).

### Discharge Criteria and Follow-Up

All patients were discharged from the hospital according to the following criteria: (a) patients were afebrile for at least 3 days, (b) respiratory symptoms improved significantly, (c) radiographic imaging showed significant improvement in acute exudative lesions, and (d) SARS-CoV-2 RT-PCR results were negative for two consecutive days. Patients in the study were required to undergo CT examinations at 3 and 6 months after discharge.

### Chest CT Scanning and Image Analysis

Chest CT scanning was performed with the patient in the supine position at end-inspiration. Patients were instructed to hold their breath to minimize motion artifacts during the procedure. All scans were performed using a uCT 760 scanner (United Imaging, Shanghai, China). The scans were acquired by using tube voltage of 120 kVp with automatic tube current. The raw data were acquired with a collimation of 0.625 mm.

Patients were assessed at admission, discharge, 3 and 6 months after discharge. A radiologist (GZ) and pulmonologist (ZL) read and analyzed the chest CT scans. Any abnormalities were recorded as items of ground-glass opacity (GGO), crazy-paving pattern, reticulation, honeycombing, parenchymal bands, consolidation, mosaicism, and bronchiectasis. The distribution of pulmonary lesions was described as random distribution, peripheral distribution, and diffuse distribution. A semi-quantitative scoring system was used to assess abnormal pulmonary involvement, which has previously been used in the pulmonary image analysis of SARS and COVID-19 (15, 20). In each of the five lung lobes, a CT severity score was assigned based on the degree of involvement: 0 point for no involvement, 1 point for <5% involvement, 2 points for 5–25% involvement, 3 points for 26–49% involvement, 4 points for 50–75% involvement, and 5 points for more than 75%. The total CT severity score was the sum of the scores of all five lung lobes, ranging from 0 (no involvement) to 25 (maximum involvement).

### Pulmonary Function Testing

During each follow-up visit, lung function tests were conducted. Pulmonary function tests were performed using a flow spirometer. The main parameters included total lung capacity (TLC), residual volume (RV), forced vital capacity (FVC), forced expiratory volume in 1 s (FEV_1_), and FEV_1_/FVC. The diffusing capacity of the lung for carbon monoxide (*D*_LCO_) was measured using the single breath method. Diffusion deficit was defined as *D*_LCO_ <80% of predicted values.

### Statistical Analysis

Statistical analyses were conducted in R version 3.5.1 (R Foundation for Statistical Computing, Vienna, Austria). Continuous variables were expressed as mean (standard deviation, SD) or medians (interquartile range, IQR), and categorical variables were expressed as frequency (*N*) and percentage (%). The difference between groups was determined using Student's *t*-test, Wilcoxon signed-rank test, or analysis of variance, or Kruskal-Wallis rank sum test, as appropriate, and Chi-squared test or Fisher's exact test for categorical variables. The linear regression model and trend test were used to evaluate the CT scores of patients. The odds ratios (ORs) and 95% confidence intervals (CIs) derived using multivariable logistic regression models were used to determine independent predictor associated with the presence of CT abnormalities. Spearman correlation method was used to assess CT score in relation to clinical variables. All statistical tests were two-sided, and a *P*-value <0.05 was considered statistically significant unless otherwise specified.

## Results

### Baseline Characteristics

In this prospective study, a total of 273 patients were recruited from February 3, 2020, to May 3, 2020 and underwent at least one CT scan at 3 and 6 months follow-up. The average age of participants was 45.48 years (SD, 15.97) and 140 (51.3%) were male (Table 1). Fifty-six patients had at least one kind of chronic disease, with approximately 13% experiencing hypertension. Of participants, 3.7% (10 of 273), 75.5% (206 of 273), 17.9% (49 of 273), and 2.9% (8 of 273) were classified as mild, moderate, severe, and critical conditions, respectively. The average time from first symptom to admission is 4.63 days. The average hospitalization period was 22.65 days (SD, 9.44 days).

**Table 1:** Clinical characteristics of 273 patients with COVID-19.

**Variables**	**Overall**
*N*	273
Age, mean (SD), y	45.48 (15.97)
**Gender**, ***N*** **(%)**
Male	140 (51.3)
Female	133 (48.7)
**Chronic disease**
**Any**, ***N*** **(%)**
No	217 (79.5)
Yes	56 (20.5)
**Hypertension**, ***N*** **(%)**
No	237 (86.8)
Yes	36 (13.2)
**Diabetes**, ***N*** **(%)**
No	260 (95.2)
Yes	13 (4.8)
**Cardiovascular disease**, ***N*** **(%)**
No	264 (96.7)
Yes	9 (3.3)
**Hepatitis B infection**, ***N*** **(%)**
No	265 (97.1)
Yes	8 (2.9)
**Cancer**, ***N*** **(%)**
No	270 (98.9)
Yes	3 (1.1)
**Disease severity**, ***N*** **(%)^a^**
Mild illness	10 (3.7)
Moderate illness	206 (75.5)
Severe illness	49 (17.9)
Critical illness	8 (2.9)
Hospitalization period, mean (SD), d	22.65 (9.44)

a *According to the Chinese clinical guidance for COVID-19 pneumonia diagnosis and treatment issued by the National Health Commission of China*.

### CT Findings at 3 and 6 Months Follow-Up

The last day of the 3 and 6 months follow-up was August 6, 2020 and September 9, 2020, respectively, with a mean interval between discharge and follow-up of 85.08 (SD, 18.09) days and 183.65 (SD, 13.03) days. A total of 957 chest CT scans was obtained throughout the study period: 273 at admission, 268 at discharge, 246 at 3 months follow-up, and 170 scans at 6 months follow-up. Two hundred forty-six of the 273 patients underwent a CT scan at 3 months follow-up (Table 2). Among them, 184 (74.8%) patients still had residual pulmonary abnormalities. Most (174 of 246, 70.7%) patients had peripheral distribution, and 123 (50%) showed bilateral lung involvement. GGO presented in over half (55.3%) of patients and parenchymal band 38.2%. Other CT manifestations included bronchiectasis, reticulation, mosaicism, consolidation, honeycombing, and crazy-paving pattern. The median of number of lobes involved was 2 (IQR, 0–4), with a median of total CT severity scores of 2 (IQR, 0–4). The CT scores of bilateral lower lobes were significantly higher than those of the corresponding upper and middle lobes (Supplementary Table 1).

**Table 2:** Findings of serial CT scans among patients with COVID-19.

	**Admission**	**Discharge**	**3 months**	**6 months**	*P*-value^*^
	**(*N* = 273)**	**(*N* = 268)**	**(*N* = 246)**	**(*N* = 170)**	
Distribution of lesions, *N* (%)					<0.001
No lesion	12 (4.4)	14 (5.2)	62 (25.2)	40 (23.5)	
Random	12 (4.4)	1 (0.4)	8 (3.3)	1 (0.6)	
Peripheral	237 (86.8)	242 (90.3)	174 (70.7)	128 (75.3)	
Diffuse	12 (4.4)	11 (4.1)	2 (0.8)	1 (0.6)	
Involvement of the lesions, *N* (%)					<0.001
No involvement	12 (4.4)	15 (5.6)	62 (25.2)	40 (23.5)	
Single lobe	46 (16.8)	46 (17.2)	61 (24.8)	45 (26.5)	
Bilateral multilobe	215 (78.8)	207 (77.2)	123 (50.0)	85 (50.0)	
Mosaicism, *N* (%)					0.151
No	268 (98.2)	268 (100.0)	243 (98.8)	169 (99.4)	
Yes	5 (1.8)	0 (0.0)	3 (1.2)	1 (0.6)	
Ground-glass opacity, *N* (%)					<0.001
No	20 (7.3)	30 (11.2)	110 (44.7)	88 (51.8)	
Yes	253 (92.7)	238 (88.8)	136 (55.3)	82 (48.2)	
Crazy-paving pattern, *N* (%)					<0.001
No	220 (80.6)	264 (98.5)	245 (99.6)	169 (99.4)	
Yes	53 (19.4)	4 (1.5)	1 (0.4)	1 (0.6)	
Reticulation, *N* (%)					0.878
No	266 (97.4)	260 (97.0)	239 (97.2)	167 (98.2)	
Yes	7 (2.6)	8 (3.0)	7 (2.8)	3 (1.8)	
Parenchymal band, *N* (%)					<0.001
No	251 (91.9)	204 (76.1)	152 (61.8)	94 (55.3)	
Yes	22 (8.1)	64 (23.9)	94 (38.2)	76 (44.7)	
Consolidation, *N* (%)					<0.001
No	245 (89.7)	247 (92.2)	244 (99.2)	169 (99.4)	
Yes	28 (10.3)	21 (7.8)	2 (0.8)	1 (0.6)	
Bronchiectasis, *N* (%)					0.484
No	266 (97.4)	260 (97.0)	234 (95.1)	163 (95.9)	
Yes	7 (2.6)	8 (3.0)	12 (4.9)	7 (4.1)	
Honeycombing, *N* (%)					0.919
No	272 (99.6)	266 (99.3)	244 (99.2)	169 (99.4)	
Yes	1 (0.4)	2 (0.7)	2 (0.8)	1 (0.6)	
No. of lobes involved, median (IQR)	4 (2, 5)	4 (2, 5)	2 (0, 4)	2 (1, 4)	<0.001
Total CT score, median (IQR)	5 (3, 9)	4 (2, 6)	2 (0, 4)	2 (1, 4)	<0.001
Left upper lobe	1 (0, 2)	1 (0, 1)	0 (0, 1)	0 (0, 1)	<0.001
Left lower lobe	2 (1, 2)	1 (1, 2)	1 (0, 1)	0 (0, 1)	<0.001
Right upper lobe	1 (0, 1)	1 (0, 1)	0 (0, 1)	0 (0, 1)	<0.001
Right middle lobe	1 (0, 1)	1 (0, 1)	0 (0, 1)	0 (0, 1)	<0.001
Right lower lobe	2 (1, 3)	1 (1, 2)	1 (0, 1)	1 (0, 1)	<0.001

* *P-values were calculated with Chi-squared test or Fisher's exact test for categorical variable, Kruskal-Wallis rank sum test for continuous variables*.

One hundred seventy patients underwent CT scans at 6 months follow-up (Table 2). Pulmonary abnormalities were observed in 130 (76.5%) patients. Most chest CT scans showed peripheral lesions and bilateral lung involvement. The most common CT findings were GGO (48.2%) and parenchymal band (44.7%). The median of number of lobes involved and total CT severity scores was 2 (IQR, 1–4) and 2 (IQR, 1–4), respectively. The CT scores of the bilateral lower lobes were significantly higher than those of upper lobes (Supplementary Table 1).

### Dynamic Changes Identified in the CT Scans

**Figure 1:**
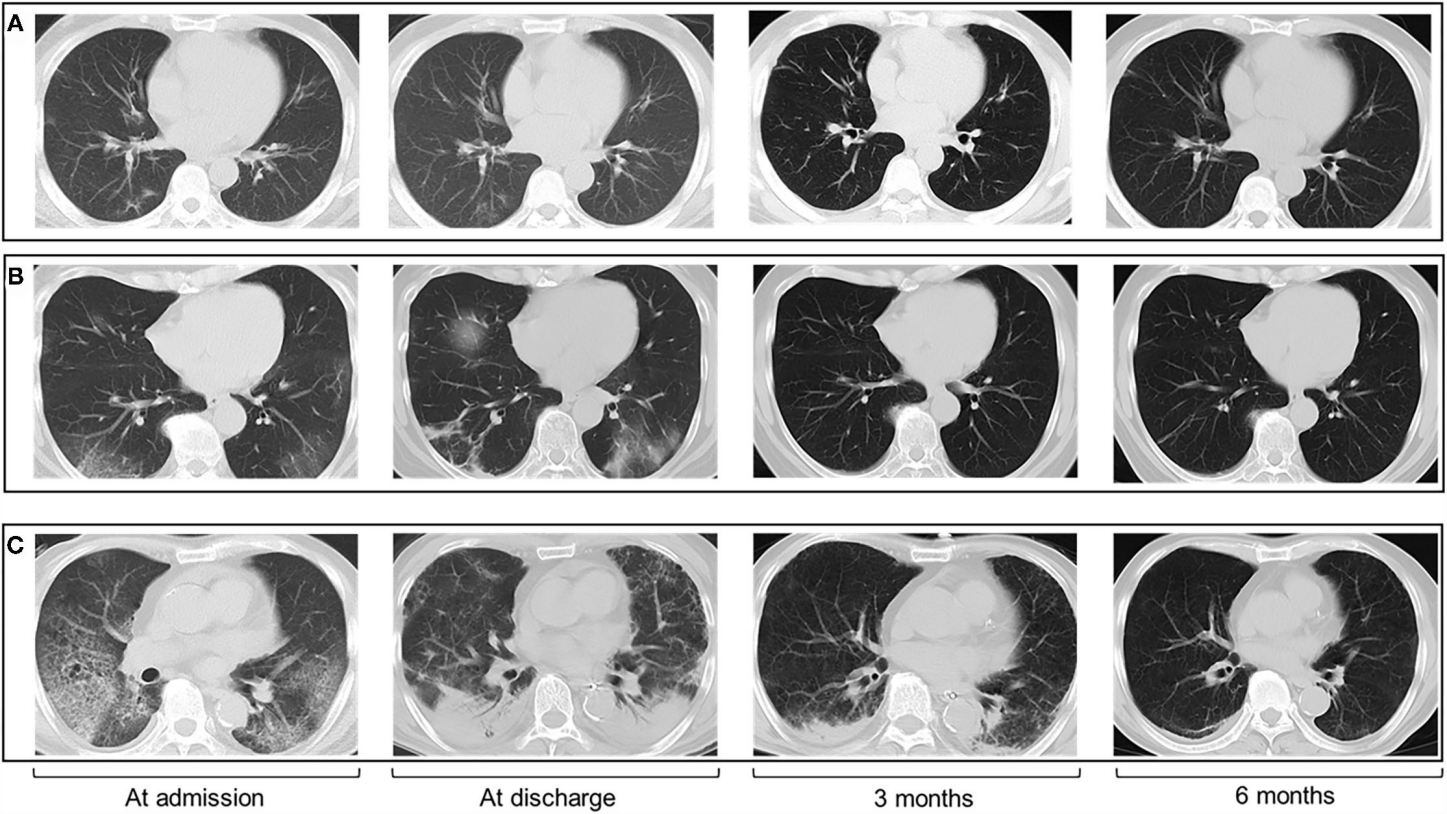
Chest CT images of patients with COVID-19 at four timepoints (admission, discharge, 3 months, and 6 months follow-up). The CT scans show GGO with bilateral lung involvement at admission and patients with greater disease severity had more residual CT abnormalities. **(A)** CT scans in a 48-year-old man with moderate illness. **(B)** CT scans in a 55-year-old woman with severe illness. **(C)** CT scans in a 67-year-old man with critical illness.

Throughout the course of the disease, chest CT scans showed bilateral lesions with peripheral distribution in most patients (Table 2; Figure 1). Ground-glass opacity was the most commonly seen abnormalities, with a gradual decrease over time (92.7% at admission, 55.3% at 6 months follow-up). Crazy-paving pattern was the second (19.4%) most common abnormality at admission, though this significantly decreased at discharge (1.5%), and became rare at 3 months (0.4%) and 6 months (0.6%) follow-up. Of note, the percentage of patients with parenchymal band increased significantly from 8.1% (22 of 273) at admission, to 23.9% (64 of 268) at discharge, to 38.2% (94 of 246) at 3 months follow-up, and 44.7% (76 of 170) at 6 months follow-up.

The total CT severity scores and the number of lobes involved were compared at the four time points (Table 2). Overall, the total CT severity scores decreased from the time of admission to the time of discharge, and thereafter at 3 months follow-up and 6 months follow-up. The number of lobes involved gradually decreased over time.

### Chest CT Findings by Disease Severity

At 3 months follow-up (Supplementary Table 2), the percentage of GGO in patients with severe or critical illness was 81.8% (45 of 55), which was significantly higher than those with moderate illness (50.0%, 91 of 182), and mild illness (0%, 0 of 9) (*P* < 0.001). Parenchymal band was found in 1 of 9 (11.1%) mild cases, 63 of 182 (34.6%) moderate cases, and 30 of 55 (54.5%) severe or critical cases, respectively. The trend test showed that the total CT score gradually increased from mild conditions to worse conditions (trend test *P* < 0.001). There was a an increase in CT score and the number of lobes involved by disease severity.

At 6 months follow-up (Supplementary Table 3), the percentage of GGO in patients with severe or critical illness was 78.4% (29 of 37), which was significantly higher than those with moderate illness (40.0%, 52 of 130), and mild illness (0%, 0 of 3). The parenchymal band was found in 0 of 3 (0%) mild cases, 60 of 130 (46.2%) moderate cases, and 16 of 37 (43.2%) severe or critical cases, respectively. The average CT score and the number of lobes involved increased significantly by disease severity.

### Dynamic Changes in CT Patterns by Disease Severity

**Figure 2:**
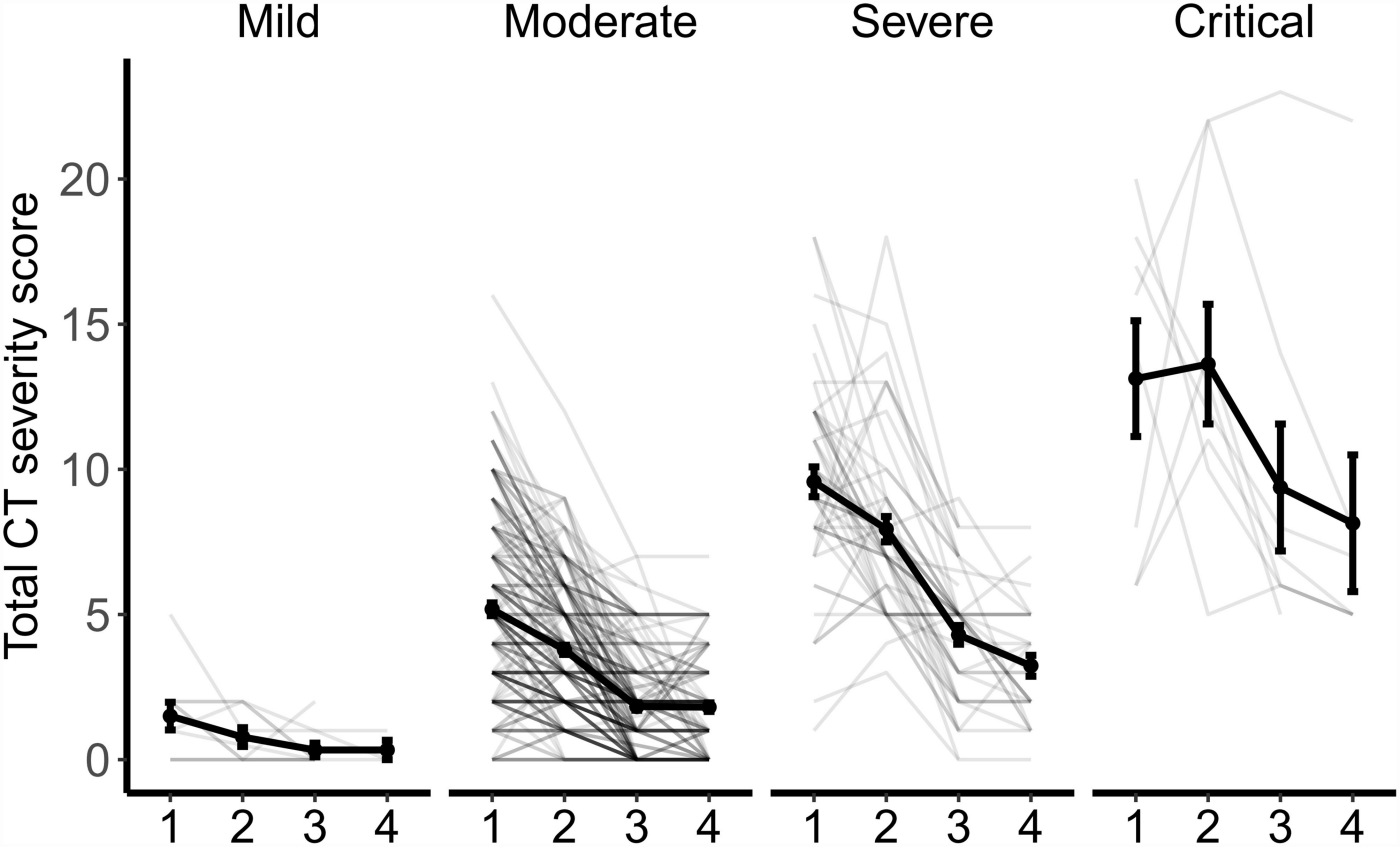
Dynamic Changes in CT scores and the number of lobes involved by disease severity. The point and error bar show the average value and standard error of total CT score at four timepoints from admission (1), to discharge (2), to 3 months (3), and 6 months (4) follow-up.

The changes in CT patterns by disease severity are shown in Figure 2. Although there was a significant decrease in CT score and the number of lobes involved, the absorption of lesions presented differently. In patients with mild conditions, the total CT score was consistently low at all four time points. The degree of lung involvement was slightly higher in moderate cases, with the mean CT value decreasing from 5.18 at admission to 1.81 at 6 months follow-up. In patients with severe conditions, the mean CT score was 9.57 at admission, 7.94 at discharge, 4.30 at 3 months follow-up, and 3.23 at 6 months follow-up. In patients with critical conditions, the residual lung lesions were the most severe, with a mean CT score of 13.13 at admission, 13.63 at discharge, 9.38 at 3 months follow-up, and 8.14 at 6 months follow-up.

### Association of Clinical Characteristics and CT Abnormalities

The association between clinical characteristics and the presence of CT abnormalities are summarized in Supplementary Table 4 and Table 3. At 3 months follow-up, there were significant differences were between the two groups by age (*P* < 0.001), any chronic disease (*P* = 0.044), hospitalization period (*P* = 0.004), and disease severity (*P* < 0.001). Multivariable analysis suggested that age (OR = 1.07; 95% CI, 1.05–1.11; *P* < 0.001) and disease severity (OR = 5.08; 95% CI, 1.34–33.36; *P* = 0.037) were independent risk factors associated with the presence of CT abnormalities at 3 months follow-up (Table 3). At 6 months follow-up, significant differences of age (*P* = 0.002), hospitalization period (*P* = 0. 001) and disease severity (*P* < 0.001) between the two groups were identified (Supplementary Table 4). Significant association between age and the risk of CT abnormalities (OR = 1.07; 95% CI, 1.03–1.11; *P* < 0.001) were observed at 6 months follow-up, and disease severity had a borderline significant effect (OR = 7.65; 95% CI, 1.40–143.02; *P* = 0.057; Table 3). Diffusion deficit was observed in 39.2% (20 of 51) of patients at 3 months and 44.4% (44 of 99) of patients at 6 months (Table 4). FVC, FEV_1_, TLC, and RV were less common. Correlation analyses between CT scores and pulmonary function testing were performed (Supplementary Figure 1). At 3 months, the CT scores were negatively correlated with TLC and RV. At 6 months follow-up, no significant correlation was found between CT scores and pulmonary function testing.

**Table 3:** Multivariable analysis of risk factors for CT abnormalities.

	**Abnormalities at 3 months**		**Abnormalities at 6 months**	
	OR (95% CI)^*^	***P*-value**	OR (95% CI)^*^	***P*-value**
Age	1.07 (1.05–1.11)	<0.001	1.07 (1.03–1.11)	<0.001
Gender		0.73		0.626
Male	1.00 (ref)		1.00 (ref)	
Female	1.12 (0.58–2.19)		0.81 (0.35–1.87)	
Any chronic disease		0.264		0.626
No	1.00 (ref)		1	
Yes	0.55 (0.19–1.64)		0.44 (0.14–1.40)	
Disease severity		0.037		0.057
Non-severe	1.00 (ref)		1.00 (ref)	
Severe	5.08 (1.34–33.36)		7.65 (1.40–143.02)	
Hospitalization period	1.03 (0.99–1.08)	0.205	1.06 (1.00–1.13)	0.074

* *Odd ratios were calculated by multivariable logistic regression models adjusting for age, gender, any chronic disease, disease severity, and hospitalization period*.

**Table 4:** Pulmonary function of COVID-19 patients at follow-up.

**Variables**	**3 months**	**6 months**
**Spirometry**	*N* = 118	*N* = 96
FVC <80% predicted, *N* (%)	4 (3.4)	6 (6.2)
FEV_1_ <80% predicted, *N* (%)	6 (5.1)	9 (9.4)
MMEF <65% predicted, *N* (%)	33 (28.4)	28 (29.2)
**Diffusion capacity**	*N* = 51	*N* = 99
*D*_LCO_ <80%, predicted, *N* (%)	20 (39.2)	44 (44.4)
*D*_LCO_ / *V*_A_ <80%, predicted, *N* (%)	11 (22.0)	21 (21.9)
**Lung volume**	*N* = 51	*N* = 99
TLC <80%, predicted, *N* (%)	6 (11.8)	14 (14.1)
RV <80% predicted, *N* (%)	2 (3.9)	2 (2.0)

## Discussion

This study prospectively assessed the clinical characteristics and CT scans of patients with COVID-19. The most common CT manifestations for patients after discharge were GGO and parenchymal band at 3 and 6 months follow-up. Our results revealed that patients with greater disease severity had higher CT severity scores at follow-up.

Long-term follow-up is needed to assess possible pulmonary damages. Previous studies have described CT abnormalities of COVID-19 patients within 3 months after discharge (21–23). This study provides additional information at 6 months follow-up, and shows that the majority of patients had chest CT imaging abnormalities in the post-discharge period. Although serial CT scans showed significant improvement, 174 of 246 (74.8%) and 130 of 170 (76.5%) patients still had pulmonary abnormalities at 3 and 6 months follow-up. The predominant characteristics were GGO with bilateral lung involvement with the peripheral distribution. This finding is in accordance with the previous studies on CT features of patients during hospitalization and during short term follow-up (7, 21). Additionally, the presence of the parenchymal band was observed in 38.2 and 48.2% of study participants at 3 and 6 months follow-up. This finding was described by a follow-up study in Iran, demonstrating that the percentage of parenchymal bands was 45.5% (10 of 22) at 3 months follow-up (22). In COVID-19 patients, the evolution of lung abnormalities on chest CT scans is similar to the progression of other forms of acute lung injury caused by viral pneumonia, such as severe acute respiratory syndrome (SARS) and Middle East respiratory syndrome (MERS). In line with previous studies, our study found that GGOs were the most common abnormality at the early stages of the COVID-19 disease. Numbers of studies have reported that the hallmark of the disease progression was a mixed pattern of consolidation on the existing GGO (15–17). In the recovery stage, however, consolidations were slowly absorbed, and repaired lung signs, such as band-like opacities (parenchymal bands) appeared. In the post-recovery stage, this study suggests that the percentage of patients with parenchymal band significantly increases at 6 months follow-up. Previous experience from SARS and MERS demonstrated that radiological abnormalities improve over time, but pulmonary fibrosis may last for months or even years (24, 25). At this point, pulmonary fibrosis and its impact on pulmonary function should be evaluated in longer term follow-up of patients with COVID-19.

This study provides insights in CT features of patients with varying levels of disease severity. Almost all patients with severe or critical illness still had substantial residual lung abnormalities at 6 months follow-up. In patients with severe or critical illness, both total CT score and the lobes involved were significantly higher than those with mild and moderate illness, indicating a slower absorption with greater disease severity. This study reveals that older age and disease severity were independent factors for residual pulmonary sequela of COVID-19 pneumonia. Different follow-up strategies should be developed according to these clinical characteristics. It is recommended to conduct follow-up visits at shorter intervals for COVID-19 patients with older age or severe conditions, and perform a comprehensive assessment of persistent or emerging pulmonary sequelae.

This longitudinal study assesses the dynamic changes in pulmonary sequelae of COVID-19 using CT scans up to 6 months after hospital discharge. Moreover, this study provides additional information on CT characteristics of patients with different degrees of disease severity, indicating that patients with severe and critical conditions need to be closely monitored for longer periods of time. There are several limitations to this study. First, this study is limited to a single-center in China. Further analysis is needed to analyze the CT findings of patients carrying epidemic strains in other regions. Second, although the CT characteristics of patients at admission and discharge are reported, these data do not reflect dynamic changes in CT findings during hospitalization. Third, because of the small number of patients undergoing pulmonary function testing during the follow-up period, the comparison of CT imaging and pulmonary function testing may be biased.

## Conclusions

In conclusion, this study shows that most patients after hospital discharge from COVID-19 still had residual pulmonary abnormalities on CT scans at 3 and 6 months of follow-up. This study provides a reference for the management of patients after hospital discharge, suggesting that long-term follow-up of chest CT scans are needed and these patients with residual pulmonary damage may need specific rehabilitation program.

## Data Availability Statement

The original contributions presented in the study are included in the article/Supplementary Material, further inquiries can be directed to the corresponding author/s.

## Ethics Statement

The studies involving human participants were reviewed and approved by Ethics Committee of Shenzhen Third People's Hospital (2020-235). The patients/participants provided their written informed consent to participate in this study.

## Author Contributions

ZZ and LL conceptualized and designed the study and reviewed and revised the manuscript. XL, DL, and ZL designed the data collection instruments, performed data analyses, and drafted the initial manuscript. ZM, LZ, JD, YS, XG, GZ, LH, LY, JC, DS, and XY collected data, performed clinical examination, carried out the initial analyses, and reviewed and revised the manuscript. QH and GL coordinated and supervised data collection and critically reviewed the manuscript for important intellectual content. All authors approved the final manuscript as submitted and agreed to be accountable for all aspects of the work.

## Funding

This work was supported by the National Science Fund for Distinguished Young Scholars (82025022), the Science and Technology Innovation Committee of Shenzhen Municipality (2020A1111350032), Bill & Melinda Gates Foundation, Sanming Project of Medicine in Shenzhen (SZSM201612025 and SZSM201911009), and Novel Coronavirus prevention and control project for Guangdong province (2020B1111340030 and 2020B1111340026).

## Conflict of Interest

The authors declare that the research was conducted in the absence of any commercial or financial relationships that could be construed as a potential conflict of interest.

## Publisher's Note

All claims expressed in this article are solely those of the authors and do not necessarily represent those of their affiliated organizations, or those of the publisher, the editors and the reviewers. Any product that may be evaluated in this article, or claim that may be made by its manufacturer, is not guaranteed or endorsed by the publisher.
