# Supplementary material for: Pulmonary Sequelae in Patients After Recovery From Coronavirus Disease 2019: A Follow-Up Study With Chest CT
Source: Front Med (Lausanne). 2022 Jan 13;8:686878. doi: 10.3389/fmed.2021.686878 (PMC8794727; doi:10.3389/fmed.2021.686878)
Supplement: Supplementary file 1 [file Data_Sheet_1.docx]

Supplementary Material

# Supplementary Tables and Figures

## Supplementary Tables

**Supplementary Table 1**. Difference of the means of CT severity score between the lower lobe, the middle lobe and the upper lobe

|  | **Difference (95% CI)** | **P value ^*^** |
| --- | --- | --- |
| **3 months follow-up** |  |  |
| Lower lobe vs. middle lobe | 0.19 (0.07, 0.32) | 0.001 |
| Lower lobe vs. upper lobe | 0.19 (0.06, 0.31) | 0.001 |
| Upper lobe vs. middle lobe | 0.01 (-0.12, 0.32) | 0.993 |
| **6 months follow-up** |  |  |
| Lower lobe vs. middle lobe | 0.12 (0.02, 0.27) | 0.113 |
| Lower lobe vs. upper lobe | 0.16 (0.01, 0.30) | 0.028 |
| Upper lobe vs. middle lobe | -0.04 (-0.18, 0.11) | 0.836 |

^*^ P value was adjusted by the Tukey HSD test

**Supplementary Table 2**. CT findings among COVID-19 patients with different disease severity at three months follow-up

|  | **Mild** | **Moderate** | **Severe / Critical** | **P value** |
| --- | --- | --- | --- | --- |
|  | **(N = 9)** | **(N = 182)** | **(N = 55)** |  |
| Distribution of lesions, N (%) |  |  |  | <0.001 |
| No lesion | 7 (77.8) | 53 (29.1) | 2 (3.6) |  |
| Random | 0 (0.0) | 5 (2.7) | 3 (5.5) |  |
| Peripheral | 2 (22.2) | 124 (68.1) | 48 (87.3) |  |
| Diffuse | 0 (0.0) | 0 (0.0) | 2 (3.6) |  |
| Involvement of the lesions, N (%) |  |  |  | <0.001 |
| No involvement | 7 (77.8) | 53 (29.1) | 2 (3.6) |  |
| Single lobe | 1 (11.1) | 55 (30.2) | 5 (9.1) |  |
| Bilateral multilobe | 1 (11.1) | 74 (40.7) | 48 (87.3) |  |
| Mosaicism, N (%) |  |  |  | 0.862 |
| No | 9 (100.0) | 180 (98.9) | 54 (98.2) |  |
| Yes | 0 (0.0) | 2 (1.1) | 1 (1.8) |  |
| Ground-glass opacity, N (%) |  |  |  | <0.001 |
| No | 9 (100.0) | 91 (50.0) | 10 (18.2) |  |
| Yes | 0 (0.0) | 91 (50.0) | 45 (81.8) |  |
| Crazy-paving pattern, N (%) |  |  |  | 0.175 |
| No | 9 (100.0) | 182 (100.0) | 54 (98.2) |  |
| Yes | 0 (0.0) | 0 (0.0) | 1 (1.8) |  |
| Reticulation, N (%) |  |  |  | <0.001 |
| No | 9 (100.0) | 182 (100.0) | 48 (87.3) |  |
| Yes | 0 (0.0) | 0 (0.0) | 7 (12.7) |  |
| Parenchymal band, N (%) |  |  |  | 0.007 |
| No | 8 (88.9) | 119 (65.4) | 25 (45.5) |  |
| Yes | 1 (11.1) | 63 (34.6) | 30 (54.5) |  |
| Consolidation, N (%) |  |  |  | 0.03 |
| No | 9 (100.0) | 182 (100.0) | 53 (96.4) |  |
| Yes | 0 (0.0) | 0 (0.0) | 2 (3.6) |  |
| Bronchiectasis, N (%) |  |  |  | 0.225 |
| No | 9 (100.0) | 175 (96.2) | 50 (90.9) |  |
| Yes | 0 (0.0) | 7 (3.8) | 5 (9.1) |  |
| Honeycombing, N (%) |  |  |  | 0.03 |
| No | 9 (100.0) | 182 (100.0) | 53 (96.4) |  |
| Yes | 0 (0.0) | 0 (0.0) | 2 (3.6) |  |
| No. of lobes involved, median (IQR) | 0 (0, 0) | 2 (0, 3) | 5 (3, 5) | <0.001 |
| Total CT score, median (IQR) | 0 (0, 0) | 2 (0, 3) | 5 (3, 6) | <0.001 |
| Left upper lobe | 0 (0, 0) | 0 (0, 1) | 1 (1, 1) | <0.001 |
| Left lower lobe | 0 (0, 0) | 0 (0, 1) | 1 (1, 1) | <0.001 |
| Right upper lobe | 0 (0, 0) | 0 (0, 1) | 1 (1, 1) | <0.001 |
| Right middle lobe | 0 (0, 0) | 0 (0, 1) | 1 (1, 1) | <0.001 |
| Right lower lobe | 0 (0, 0) | 0 (0, 1) | 1 (1, 1) | <0.001 |

* P values were calculated with Chi-squared test or Fisher's exact test for categorical variable, Kruskal-Wallis rank sum test for continuous variables.

**Supplementary Table 3**. CT findings among COVID-19 patients with different disease severity at six months follow-up

|  | **Mild** | **Moderate** | **Severe / Critical** | **P value *** |
| --- | --- | --- | --- | --- |
|  | **(N = 3)** | **(N = 130)** | **(N = 37)** |  |
| Distribution of lesions, N (%) |  |  |  | 0.008 |
| No lesion | 2 (66.7) | 37 (28.5) | 1 (2.7) |  |
| Random | 0 (0.0) | 1 (0.8) | 0 (0.0) |  |
| Peripheral | 1 (33.3) | 92 (70.8) | 35 (94.6) |  |
| Diffuse | 0 (0.0) | 0 (0.0) | 1 (2.7) |  |
| Involvement of the lesions, N (%) |  |  |  | <0.001 |
| No involvement | 2 (66.7) | 37 (28.5) | 1 (2.7) |  |
| Single lobe | 1 (33.3) | 38 (29.2) | 6 (16.2) |  |
| Bilateral multilobe | 0 (0.0) | 55 (42.3) | 30 (81.1) |  |
| Mosaicism (%) |  |  |  | 0.164 |
| No | 3 (100.0) | 130 (100.0) | 36 (97.3) |  |
| Yes | 0 (0.0) | 0 (0.0) | 1 (2.7) |  |
| Ground-glass opacity, N (%) |  |  |  | <0.001 |
| No | 2 (66.7) | 78 (60.0) | 8 (21.6) |  |
| Yes | 1 (33.3) | 52 (40.0) | 29 (78.4) |  |
| Crazy-paving pattern, N (%) |  |  |  | 0.164 |
| No | 3 (100.0) | 130 (100.0) | 36 (97.3) |  |
| Yes | 0 (0.0) | 0 (0.0) | 1 (2.7) |  |
| Reticulation, N (%) |  |  |  | 0.004 |
| No | 3 (100.0) | 130 (100.0) | 34 (91.9) |  |
| Yes | 0 (0.0) | 0 (0.0) | 3 (8.1) |  |
| Parenchymal band, N (%) |  |  |  | 0.277 |
| No | 3 (100.0) | 70 (53.8) | 21 (56.8) |  |
| Yes | 0 (0.0) | 60 (46.2) | 16 (43.2) |  |
| Consolidation, N (%) |  |  |  | 0.857 |
| No | 3 (100.0) | 129 (99.2) | 37 (100.0) |  |
| Yes | 0 (0.0) | 1 (0.8) | 0 (0.0) |  |
| Bronchiectasis, N (%) |  |  |  | 0.372 |
| No | 3 (100.0) | 126 (96.9) | 34 (91.9) |  |
| Yes | 0 (0.0) | 4 (3.1) | 3 (8.1) |  |
| Honeycombing, N (%) |  |  |  | 0.164 |
| No | 3 (100.0) | 130 (100.0) | 36 (97.3) |  |
| Yes | 0 (0.0) | 0 (0.0) | 1 (2.7) |  |
| No. of lobes involved, median (IQR) | 0 (0, 1) | 2 (0, 3) | 4 (2, 5) | <0.001 |
| Total CT score, median (IQR) | 0 (0, 1) | 2 (0, 3) | 4 (2, 5) | <0.001 |
| Left upper lobe | 0 (0, 0) | 0 (0, 1) | 1 (0, 1) | 0.001 |
| Left lower lobe | 0 (0, 0) | 0 (0, 1) | 1 (1, 1) | <0.001 |
| Right upper lobe | 0 (0, 1) | 0 (0, 1) | 1 (0, 1) | <0.001 |
| Right middle lobe | 0 (0, 0) | 0 (0, 1) | 1 (0, 1) | 0.029 |
| Right lower lobe | 0 (0, 0) | 0 (0, 1) | 1 (1, 1) | <0.001 |

* P values were calculated with Chi-squared test or Fisher's exact test for categorical variable, Kruskal-Wallis rank sum test for continuous variables.

**Supplementary Table 4**. Association of clinical characteristics and CT abnormalities

|  | **Abnormalities at 3 months** | | |  | **Abnormalities at 6 months** | | |
| --- | --- | --- | --- | --- | --- | --- | --- |
|  | **No** | **Yes** | **P value** |  | **No** | **Yes** | **P value** |
| N | 62 | 184 |  |  | 40 | 130 |  |
| Age, mean (SD), y | 34.58 (13.22) | 49.84 (14.30) | <0.001 |  | 39.30 (15.55) | 50.80 (12.28) | <0.001 |
| Gender, N (%) |  |  | 1 |  |  |  | 0.974 |
| Male | 32 (51.6) | 94 (51.1) |  |  | 22 (55.0) | 69 (53.1) |  |
| Female | 30 (48.4) | 90 (48.9) |  |  | 18 (45.0) | 61 (46.9) |  |
| Any chronic disease, N (%) |  |  | 0.044 |  |  |  | 0.471 |
| No | 55 (88.7) | 139 (75.5) |  |  | 33 (82.5) | 98 (75.4) |  |
| Yes | 7 (11.3) | 45 (24.5) |  |  | 7 (17.5) | 32 (24.6) |  |
| Disease severity, N (%) |  |  | <0.001 |  |  |  | 0.002 |
| Non-severe | 60 (96.8) | 131 (71.2) |  |  | 39 (97.5) | 94 (72.3) |  |
| Severe | 2 (3.2) | 53 (28.8) |  |  | 1 (2.5) | 36 (27.7) |  |
| Hospitalization period, mean (SD), d | 19.97 (7.45) | 24.03 (9.97) | 0.004 |  | 19.05 (7.01) | 24.62 (9.88) | 0.001 |

## Supplementary Figures


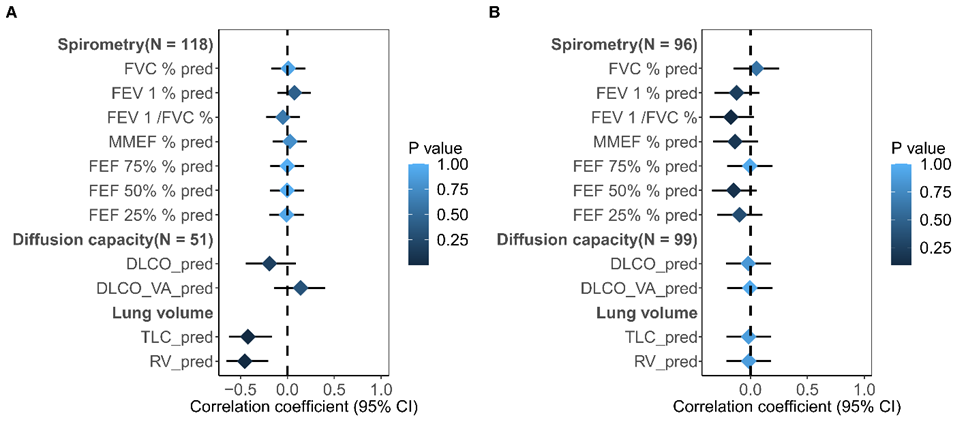


**Supplementary Figure 1.** **Association of CT scores and pulmonary function testing**. The forest plot shows Pearson correlation coefficient (95% CI) between CT scores and pulmonary function testing at (A) 3 months and (B) 6 months follow-up.
